# IgG Antibody Response to the Pfizer BNT162b2 SARS-CoV-2 Vaccine in Healthcare Workers with Healthy Weight, Overweight, and Obesity

**DOI:** 10.3390/vaccines10040512

**Published:** 2022-03-25

**Authors:** John T. Bates, Andrew P. Farmer, Michael A. Bierdeman, Dallas R. Ederer, Lauren S. Carney, Denise D. Montgomery, Seth T. Lirette, Gailen D. Marshall

**Affiliations:** 1 Department of Microbiology & Immunology, University of Mississippi Medical Center, Jackson, MS 39216, USA; 2 Department of Medicine, University of Mississippi Medical Center, Jackson, MS 39216, USA; apfarmer@umc.edu (A.P.F.); mbierdeman@umc.edu (M.A.B.); ddmontgomery@umc.edu (D.D.M.); gmarshall@umc.edu (G.D.M.); 3 Medical Student Research Program, University of Mississippi Medical Center, Jackson, MS 39216, USA; dederer@umc.edu (D.R.E.); lcarney@umc.edu (L.S.C.); 4 School of Population Health, University of Mississippi Medical Center, Jackson, MS 39216, USA; slirette2@umc.edu

**Keywords:** SARS-CoV-2, vaccination, obesity, body mass index, IgG, mRNA vaccine

## Abstract

Obesity is a significant factor for increased morbidity and mortality upon infection with SARS-CoV-2. Because of the higher potential for negative outcomes following infection of individuals with obesity, the impact of body mass index (BMI) on vaccine immunogenicity and efficacy is an important public health concern. Few studies have measured the magnitude and durability of the vaccine-specific response in relation to BMI. We measured the receptor binding domain (RBD)-specific serum IgG and surrogate neutralizing titers in a cohort of 126 vaccinated individuals with no clinical history or serological evidence of previous SARS-CoV-2 infection 50 and 200 days following vaccination. BMI had no significant impact on RBD-specific IgG titers and surrogate neutralizing titers 50 days following immunization, and leptin levels had no correlation with the response to immunization. Two hundred days following immunization, antibody titers in all groups had declined by approximately 90%. The responses were also similar between male and female participants and did not significantly vary across age groups. These results indicate that the magnitude and durability of the antibody response to mRNA-based vaccines are unaffected by BMI in this cohort.

## 1. Introduction

Approximately 12% of the world adult population has obesity, and obesity rates in some countries exceed 30% [1]. Obesity and health problems associated with obesity are significant risk factors for COVID-19 [2,3,4,5,6,7,8], making individuals with obesity an at-risk population. These individuals have lower titers of SARS-CoV-2-specific antibodies following infection [9] and those antibodies are less effective at neutralizing SARS-CoV-2 [10].

The recently approved mRNA vaccines are highly effective at reducing infection and morbidity in the general population [11,12] and have been recommended for individuals with obesity [13]. However, the efficacy and durability of mRNA vaccine-specific responses in individuals with overweight and obesity remains unknown. In adults, high BMI has been negatively correlated with the response to vaccination against hepatitis B [14,15], rabies [16], and influenza [17,18,19]. However, other studies have found that obesity does not significantly affect the response to vaccination [20,21]. The impact of obesity on mRNA-based SARS-CoV-2 vaccines remains an open question. One report has found that healthy weight individuals have higher antibody titers than individuals in the pre-obesity group 21 days following vaccination, though individuals with obesity did not have significantly different titers from normal-weight individuals [22]. A second study by the same group found similar antibody responses among normal weight, pre-obesity, and obesity groups [23].

We measured the SARS-CoV-2 spike protein-specific IgG and surrogate neutralizing responses in vaccinated health care workers approximately 50 and 200 days following vaccination and found that the magnitude and durability of the RBD-specific antibody response was unaffected by BMI or serum leptin levels among members of this cohort.

## 2. Materials and Methods

### 2.1. Study Design and Cohort

The samples analyzed for this study were taken from a cohort of 202 individuals who are current or former employees of the University of Mississippi Medical Center (UMMC). The study was approved by the UMMC IRB, and informed consent was obtained from all subjects involved in the study. Samples from individuals with a previous diagnosis of SARS-CoV-2 (CoV-2) infection or with serological evidence of infection, e.g., SARS-CoV-2 nucleocapsid (NP)-specific IgM or IgG in serum, were excluded from this analysis. Samples from individuals diagnosed with cancer currently undergoing chemotherapy were also excluded. After accounting for these exclusions, samples from 126 individuals collected approximately 50 days following the second immunization were analyzed (Table 1). All participants were immunized twice, and for all participants, the second immunization was administered approximately three weeks following the first immunization, as per the standard immunization schedule at that time. Twenty-seven individuals were lost to follow-up or experienced breakthrough infections prior to the second sample collection approximately 200 days following immunization. Data from samples with antibody profiles consistent with breakthrough infection, i.e., NP-specific antibody responses or increased RBD-specific responses relative to the day 50 time point, were excluded from the day 200 data set. All samples were collected prior to FDA approval booster vaccinations.

### 2.2. Recombinant Antigens and ELISA

Recombinant RBD protein bearing a 6x histidine tag was expressed in Expi293 cells (ThermoFisher, Waltham, MA, USA) from a construct synthesized by Twist Biosciences that encodes amino acids 319–542 of the SARS-CoV-2 spike protein. Expi293 cells were transfected with plasmid and grown for six days. Culture supernatant was harvested and passed over a HisTrap HP column (Cytiva, Marlborough, MA, USA). RBD protein was eluted with 450 mM imidazole. Imidazole was removed via buffer exchange using a centrifugal filter device with a 10,000 daltons cutoff (Pall Corp., Port Washington, NY, USA). Antibodies specific for the receptor binding domain (RBD) of the SARS-CoV-2 spike protein were measured by ELISA as previously described [24]. All reactions were performed in triplicate. Endpoint dilution titers were defined as the inverse of the highest dilution that resulted in an absorbance value of 0.2 over that of naive human sera plated at the same dilutions. Pooled human sera collected prior to the emergence of SARS-CoV-2 were considered to be naïve and used for the negative control condition.

### 2.3. Surrogate Neutralizing Antibody ELISA

As a surrogate for direct neutralization of SARS-CoV-2 virus, we determined the titers of antibodies in serum that interfered with binding of the spike RBD with recombinant hACE2 protein as previously described [25]. All reactions were performed in triplicate. Recombinant RBD used in this assay was expressed as described above and conjugated to HRP using a HRP conjugation kit (Abcam, Waltham, MA, USA).

### 2.4. Leptin ELISA

Leptin levels were measured using the Leptin ELISA kit from Bertin Bioreagent (Montigny-le-Bretonneux, France).

### 2.5. Statistical Analysis

Primary graphs were constructed using standard boxplot techniques with superimposed group means and jittered scatterplots. Pearson’s correlation coefficients were calculated where appropriate. *p*-values for differences across groups were calculated with random slope-intercept models operating under the missing-at-random assumption. All analyses were completed with Stata v17.1.

## 3. Results

### 3.1. BMI Does Not Affect Vaccine-Elicited Receptor RBD-Specific IgG Titers

**Figure 1:**
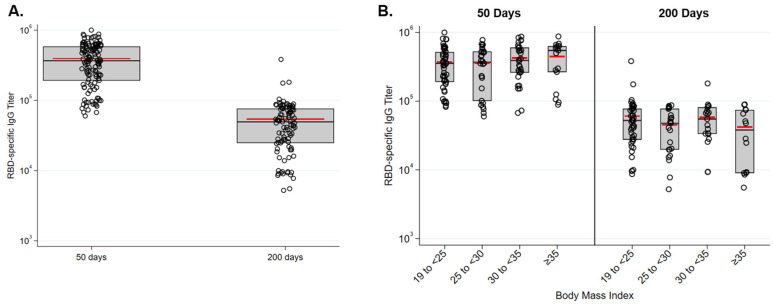
SARS-CoV-2 RBD-specific serum IgG endpoint dilution titers 50 and 200 days following vaccination with BNT162b2. (**A**) Antibody titers declined significantly between day 50 and day 200 (*p* < 0.001). (**B**) Titers did not significantly differ at either time point in relation to the participants’ BMI.

We measured RBD-specific serum IgG levels approximately 50 and 200 days following immunization (Figure 1A). Individual responses 50 days following immunization showed a great deal of heterogeneity. End point dilution titers ranged from 6 × 10^4^ to >10^6^, with an average titer of 393,000. Two hundred days following immunization, titers significantly dropped (*p* < 0.001) and ranged from 6000 to 383,000 with an average titer of 54,000. Endpoint dilution titers did not significantly vary among BMI-based groups 50 or 200 days following immunization (Figure 1B). The length of time between immunization and sample collection was approximately 50 and 200 days and did not significantly vary as a function of BMI group (Supplemental Figure S1).

### 3.2. BMI Does Not Affect Surrogate Neutralizing Antibody Activity in Sera

**Figure 2:**
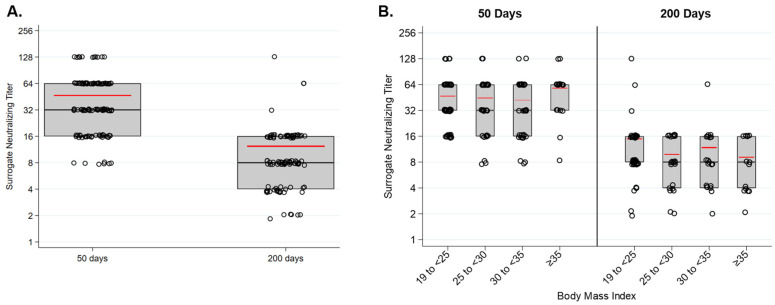
SARS-CoV-2 surrogate neutralizing titers 50 and 200 days following vaccination with BNT162b2. (**A**) Surrogate neutralizing titers significantly declined between day 50 and day 200 (*p* < 0.001). (**B**) Surrogate neutralizing titers did not significantly differ at either time point in relation to the participants’ BMI.

We also measured the ability of vaccine-elicited antibodies to interfere with binding of recombinant RBD to recombinant hACE2. Only antibodies that block binding of RBD to ACE2 are detected by this assay. Surrogate neutralizing titers decline substantially between the two time points (Figure 2A). As with total RBD-specific IgG, the surrogate neutralizing titer did not significantly vary among groups based upon BMI at either time point (Figure 2B).

### 3.3. Leptin Levels Did Not Correlate with Antibody Titers

**Figure 3:**
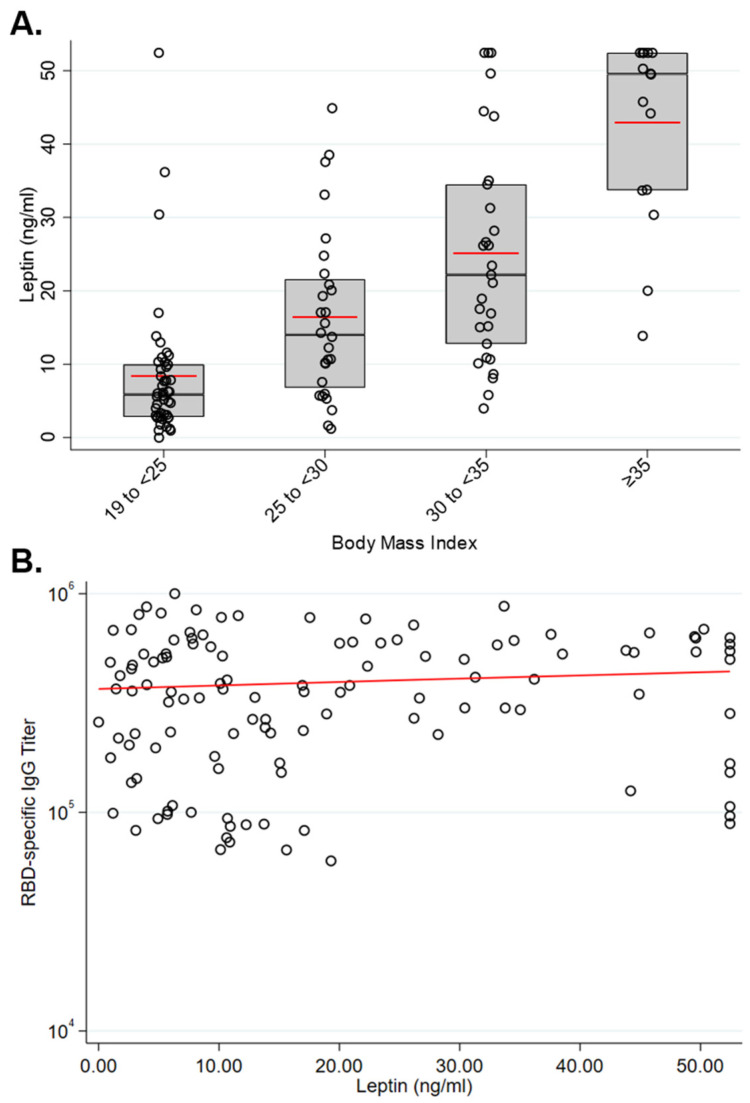
Serum leptin levels 50 days following vaccination. (**A**) Leptin levels significantly increased across BMI groups (*p* < 0.001). (**B**) Leptin levels were not significantly correlated with RBD-specific serum IgG levels 50 days following immunization (correlation = 0.10, *p* = 0.278).

While BMI is a widely accepted estimation of obesity, some individuals with overweight and obese BMI may not have excess adipose tissue relative to individuals with healthy weight BMIs. To determine if the BMI values for this cohort generally reflected the health status of study participants, we also measured serum leptin levels by ELISA in samples collected 50 days following immunization. As expected, leptin levels were significantly higher in the obese and very obese groups relative to the healthy weight groups (Figure 3A). However, leptin levels did not significantly correlate with antibody titers (Figure 3B). Leptin levels did not significantly correlate with antibody titers within BMI groups (data not shown).

### 3.4. Antibody Responses Are Similar in Females and Males and Did Not Significantly Vary across Age

**Figure 4:**
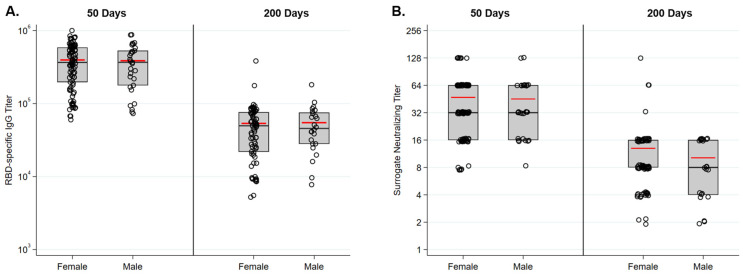
Antibody responses to vaccination are similar in males and females. (**A**) RBD-specific IgG titers as measured by ELISA were similar in females and males 50 and 200 days following immunization. (**B**) SARS-CoV-2 surrogate neutralizing titers were similar in females and males 50 and 200 days following immunization.

**Figure 5:**
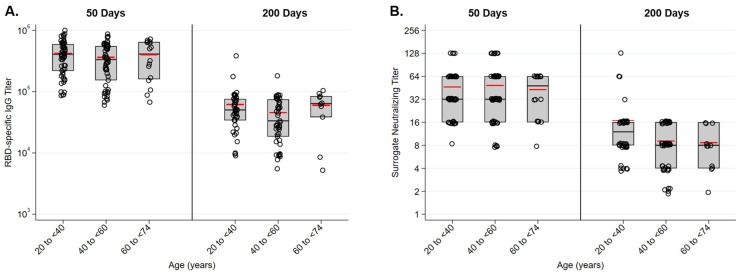
Antibody response to vaccination were similar across age groups. (**A**) RBD-specific IgG titers as measure by ELISA were similar in 20 to <40, 40 to <60, and 60 to <74 age groups. (**B**) SARS-CoV-2 surrogate neutralizing titers were similar 20 to <40, 40 to <60, and 60 to <74 age groups.

RBD-specific IgG responses following immunization were similar in males and females in this cohort (Figure 4A). Fifty days following immunization the average titer for females in the study was 397,000 and the average titer for males was 381,000. Two hundred days following immunization titers had waned to 53,000 for both groups. Surrogate neutralizing titers were also similar at both time points in females and males (Figure 4B). Antibody responses did not vary across age groups. RBD-specific IgG levels were equivalent for all age groups 50 and 200 days following immunization (Figure 5A). Surrogate neutralizing titers were also similar across all groups at both time points (Figure 5B).

## 4. Discussion

There is appropriate concern about the specificity, intensity, and duration of protective antibody levels in patients vaccinated against SARS-CoV-2 particularly in light of emerging evidence supporting needs for booster injections and new emerging strains that may not be as sensitive to current vaccine-directed responses. Concern exists that individuals with obesity are at increased risk for higher morbidity and mortality from COVID-19 [26,27]. Part of the presumed mechanism relates to assumed inferior response to the various SARS-CoV-2-specific vaccines. We have shown that, in this cohort, BMI had no significant impact on vaccine-specific antibody titers after immunization with the BNT162b2. Given the reportedly adverse correlation between obesity and morbidity following infection with SARS-CoV-2, assessing whether SARS-CoV-2-specific vaccine effectiveness may benefit from using biomarkers such as specific antibody titers in individuals with obesity may be important.

Our observations suggest that the biological mechanisms which contribute to increased morbidity and mortality suffered by individuals with obesity as a result of COVID-19 do not impair the response to vaccination in these individuals. These observations contrast with reports of reduced response to vaccination against influenza in individuals with obesity [17,18,19]. Interestingly, Malavazos and colleagues found that abdominal obesity (AO), negatively affected antibody levels in immunized individuals without a history of infection ~100 days following immunization [28], while others have reported a negative effect of BMI on antibody response among males but not females [29]. These varying results could be explained by a faster antibody decay phase in individuals with obesity followed by a similar plateau at a longer time points, ~200 days in our study. Additionally, the kinetics of RBD-specific antibodies which we report could vary from the kinetics of total spike-specific IgG. Population and environmental differences among continents are another potential explanation for the different results of these three studies.

Leptin has many immunological effects relevant to infection risk [30], including immune senescence-inducing effects on B cells [31]. However, leptin levels among participants in this study did not negatively correlate with the antibody response to vaccination. This observation is similar to previous reports that no relationship between leptin levels the antibody response to vaccination [32]. These data suggest that obesity-associated risks from SARS-CoV-2 infection are mediated by mechanisms that are not critical to the response to vaccination.

Many questions remain about the specific immune responses to BNT162b2, e.g., durability of the antibody response at more distant time points and the characteristics of the vaccine-specific T cell responses, and whether durability is affected by obesity. To date, the Pfizer vaccine is highly effective at preventing breakthrough infections, but given the relatively rapid decay of antibody-specific IgG that we observed (Figure 1 and Figure 2), the rate of breakthrough infections is likely to increase with time following vaccination. We have observed in our cohort that vaccination stimulates a RBD-specific antibody response several fold stronger than natural infection (unpublished observations), and others have reported that individuals with obesity mount a less effective antibody response to natural infection with SARS-CoV-2 [10]. Together, these findings raise the possibility that, for individuals with obesity, vaccine-mediated immunity may be more protective than immunity from primary infection at preventing reinfection and limiting morbidity upon reinfection

Antibodies are the presumed surrogate for protection against infection with most viruses [33], but the surrogate for protection for SARS-CoV-2 has not yet been established. Early studies of the human coronavirus 229E revealed that virus-specific neutralizing antibodies in serum did not protect against infection [34]. If antibodies are not the correlate of protection for SARS-CoV-2, the impact of BMI on vaccine-specific T-cell responses may be one factor associated with the ultimate success of vaccines in populations with obesity. This hypothesis will be tested in future studies.

## 5. Conclusions

The antibody response to immunization with BNT162b2 is unaffected by BMI.

## Figures and Tables

**Table 1:** Cohort BMI, sex, and age.

	Sex		Age (Years)		
BMI	Female	Male	23 to <40	40 to <60	60 to <74
19 to <25	40	9	28	18	3
25 to <30	18	12	10	16	4
30 to <35	25	5	10	14	6
≥35	14	3	7	7	3
TOTAL	97	29	55	55	16

## Data Availability

Not applicable.

## Supplementary Materials

The following supporting information can be downloaded at: https://www.mdpi.com/article/10.3390/vaccines10040512/s1, Figure S1: Time period from immunization to sample collection did not significantly vary among BMI groups.
