# Supplementary material for: IgG Antibody Response to the Pfizer BNT162b2 SARS-CoV-2 Vaccine in Healthcare Workers with Healthy Weight, Overweight, and Obesity
Source: Vaccines (Basel). 2022 Mar 25;10(4):512. doi: 10.3390/vaccines10040512 (PMC9025933; doi:10.3390/vaccines10040512)
Supplement: Supplementary file 1 [file vaccines-10-00512-s001.zip › vaccines-1631606-supplementary.pdf]

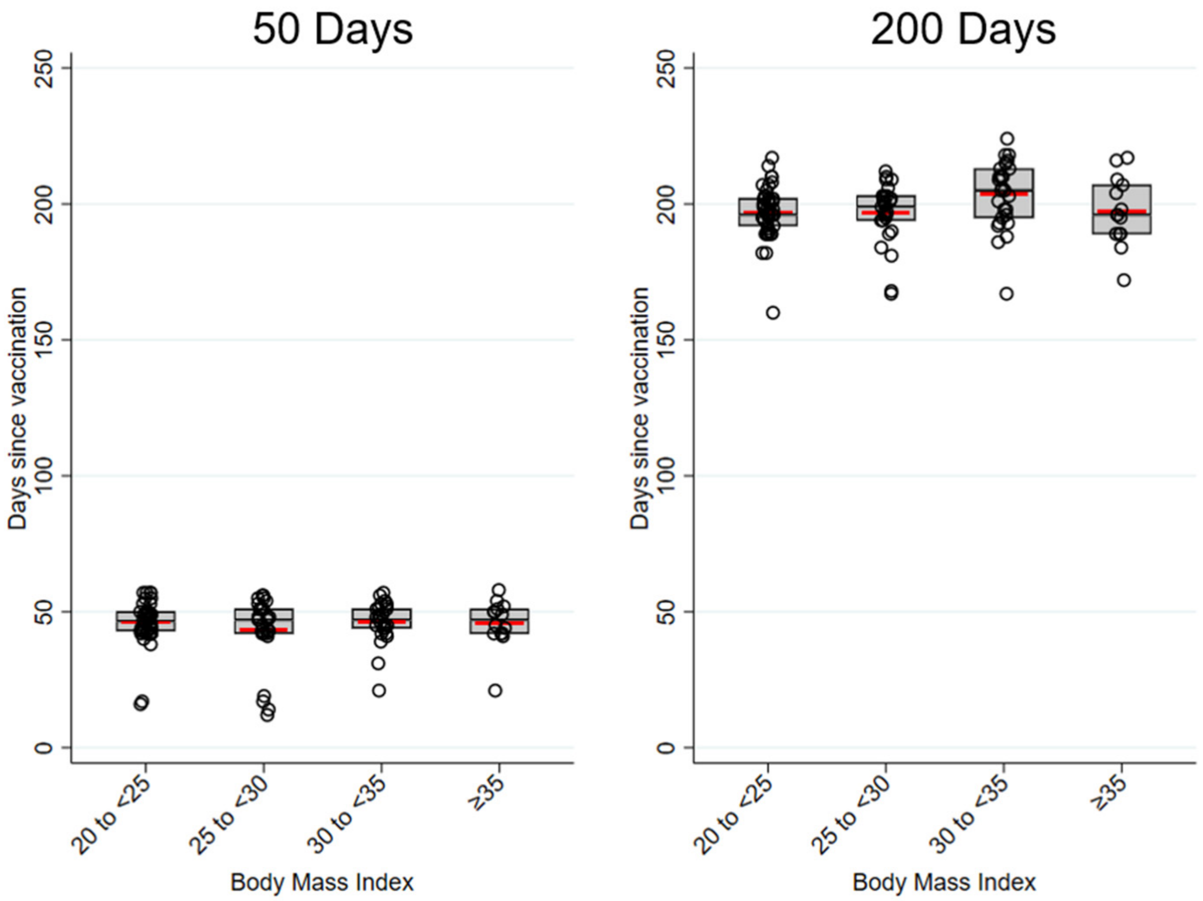

Supplemental Figure S1. Time period from immunization to sample collection did not significantly vary among BMI groups.
